# Potential Antidepressant Role of Neurotransmitter CART: Implications for Mental Disorders

**DOI:** 10.1155/2011/762139

**Published:** 2011-07-07

**Authors:** Peizhong Mao

**Affiliations:** ^1^The Division of Neuroscience, Oregon National Primate Research Center, Oregon Health and Science University, 505 NW 185th Avenue, Beaverton, OR 97006, USA; ^2^The Department of Public Health and Preventive Medicine, the Knight Cancer Institute, Oregon Health and Science University, Portland, OR 97239, USA

## Abstract

Depression is one of the most prevalent and debilitating public health concerns. Although no single cause of depression has been identified, it appears that interaction among genetic, epigenetic, biochemical, environmental, and psychosocial factors may explain its etiology. Further, only a fraction of depressed patients show full remission while using current antidepressants. Therefore, identifying common pathways of the disorder and using that knowledge to develop more effective pharmacological treatments are two primary targets of research in this field. Brain-enriched neurotransmitter CART (cocaine- and amphetamine-regulated transcript) has multiple functions related to emotions. It is a potential neurotrophic factor and is involved in the regulation of hypothalamic-pituitary-adrenal axis and stress response as well as in energy homeostasis. CART is also highly expressed in limbic system, which is considered to have an important role in regulating mood. Notably, adolescents carrying a missense mutation in the CART gene exhibit increased depression and anxiety. Hence, CART peptide may be a novel promising antidepressant agent. In this paper, we summarize recent progress in depression and CART. In particular, we emphasize a new antidepressant function for CART.

## 1. Introduction

Depression or major depression disorder (MDD) is one of the most prevalent and debilitating public health concerns. MDD affects millions of people each year [1, 2], and the burden of this disease will continue to increase, especially during the extra years of life gained from improved health outcomes in cardiovascular disease, cancer, and other domains [3, 4]. Notably evidence showed that MDD affects more women than men [5]. 

According to the guidelines developed by the American Psychiatric Association, MDD can be diagnosed when a patient demonstrates at least 2 weeks of depressed mood or loss of interest accompanied by at least four additional symptoms, including constant sadness, irritability, hopelessness, trouble sleeping, low energy or fatigue, feeling worthless or guilty for no reason, significant weight change (gain or loss), difficulty concentrating, and loss of interest in favorite activities [5, 6]. However, the etiology and pathology of this serious biologic disease are still largely unknown. It is likely the result of a complex interaction of genetic, epigenetic, biochemical, environmental, and psychosocial factors. 

Now, there is compelling evidence that monoaminergic neurotransmission in the brain is disturbed in depressed patients. However, usually it takes between 1 and 6 weeks for the current antidepressant medicines to exert their clinical effects. This latency is thought to be a problem in the therapy of MDD, since many patients have a high risk of committing suicide. Furthermore, only 50% of patients with MDD show full remission while receiving currently available antidepressants [4]. Thus, faster and more effective pharmacological treatments for MDD are greatly needed. 

The cocaine- and amphetamine-regulated transcript (CART) peptides are among the newest putative peptide neurotransmitters [7–10]. It recently has been hypothesized to be an interesting neuropeptide that might be relevant to the treatment of depression [11]. CART peptides show no significant homology to any other peptide, and as discussed below, they have unique structure, expression and multiple roles in several physiological processes. CART peptides are involved in reward and reinforcement, feeding, sensory processing, stress response, and endocrine control. CART also regulates monoaminergic neurotransmission and neurotrophic factors such as brain-derived neurotrophic factor (BDNF). Interestingly, previous research on the activity of CART established an important connection between this peptide and mitochondria, the energy producing structures in most cells [12]. More importantly, animal studies have shown an antidepressant effect for CART peptides. Therefore, CART may be a new antidepressant candidate with great promise for future clinical utility. 

## 2. Recent Progress of Depression

To date, the etiopathogenesis of MDD is still unknown. Despite the limited knowledge available to explain the cause and molecular mechanisms underlying this disease, there are several hypotheses and related effective treatments for depressed patients. 

 The widely used tricyclic compound imipramine and the antituberculosis drug iproniazid were early treatments that effectively treated depression. Both drugs cause elevation of extracellular monoamine levels by either blocking monoamine oxidase (MAO) (like iproniazid) or by inhibiting the neuronal serotonin and/or noradrenaline transporter (like imipramine and its active metabolite desipramine). These drugs' effectiveness led to the hypothesis that depression (affective disorders) is due to the central nervous system (CNS) “catecholamine deficiency”. Later, the introduction of selective serotonin reuptake inhibitors (SSRIs such as fluoxetine) drove the “monoamine deficiency” hypothesis [6, 13]. Interestingly, serotonin is thought to regulate neurodevelopmental processes through maternal-fetal interactions that have long-term mental health implications, and recently, it has been discovered that there is a placental serotonin synthetic pathway from a maternal tryptophan precursor in both mice and humans [14]. Since monoamine (including serotonin) enhancers improve depressive symptoms, the monoamine hypothesis continues to be a prominent preoccupation of the field, and the pathway of monoaminergic transmission continues to be the one of the key targets of new medications. However, many studies, including genetic association analyses examining polymorphisms in monoaminergic genes, provide little evidence to implicate true deficits in serotonergic, noradrenergic, or dopaminergic neurotransmission in the pathophysiology of depression [4, 15]. 

Beyond the serotonin hypothesis, increasing evidence strongly suggests components of mitochondrial dysfunction/oxidative stress and inflammation in the pathogenesis of depression and other affective spectrum disorders [16–18]. These three concepts of monoamines, energy metabolism, and inflammatory pathways are interrelated in many complex manners. For example, the major categories of drugs used to treat depression have also been demonstrated to exert effects on mitochondria and inflammation. Conversely, commonly used mitochondrial-targeted treatments exert effects on mitochondria and inflammation, and are increasingly being shown to demonstrate efficacy in the affective spectrum disorders [17]. These observations indicate that mitochondria protection or stimulating mitochondrial function is an important therapeutic approach for treatment of depression and related diseases. 

Over the last several years, genome-wide association studies (GWAS) have become a powerful tool for unraveling the genetic foundations of complex disorders such as major depression. A recent GWAS analysis [19] also suggests that HOMER1 plays a role in the etiology of major depression. Observations from homer1 knockout mice and human imaging genetics study indicate the importance of cortical-limbic circuitry in the development of MDD. It is of great interest that CART and Homer1 have already been implicated in a previous report [20]. In this study, microarray analysis and real-time polymerase chain reaction (PCR) showed both genes were similarly downregulated in the frontal cortex of rats exposed to the chronic mild stress paradigm, an animal model of human depression.

On the other hand, depression is frequent psychiatric disorder in neurodegenerative diseases such as Alzheimer's disease (AD) and multiple sclerosis [21–23], suggesting there is an important mechanism that neurodegeneration or neuron loss not only underlies dementia and movement disorder but also causes depression. Neuropsychiatric symptoms are common even in the early stages of AD [22]. Depression also decreases the quality of life but may remain unrecognized in AD patients [22]. Some of AD patients develop psychosis as the disease progresses. The associations of candidate genes for this type of AD have also been indicated with the monoamine neurotransmitter system and dopamine system [24]. 

Importantly, loss of cholinergic neurons and/or dysfunction of the glutamatergic system in CNS cause learning impairment in AD patients and experimental AD animals. Further, the impaired cholinergic system is likely implicated in depressive behaviors in AD patients. Neurogenesis persistently occurs in the forebrain subventricular zone (SVZ) and hippocampal subgranular zone (SGZ) in rodent and human brains. Impaired neurogenesis in those regions is implicated not only in memory deficits but also in depressive behaviors. It has been found that olfactory bulbectomized (OBX) mice reveal memory impairment and depressive behaviors [25]. A novel cognitive enhancer, ZSET1446, that is a new azaindolizinone derivative improved learning and memory in the hippocampus of amyloid-beta infused rats. Notably, ZSET1446 increased neurogenesis and improved depressive behavior [25]. These therapeutic data suggest that neurodegeneration or impaired neurogenesis in the brain may be an important and common mechanism of the depression and learning/memory/cognitive impairment observed in AD animal models and AD patients.

Furthermore, neurotrophins are important regulators of neurogenesis and neuronal plasticity in the developing and adult brain. Neurotrophin signalling has also been strongly linked in mood disorders. Recent studies on neurotrophic factors, in particular BDNF, have indicated that mental disorders reflect failed function of critical neuronal networks, whereas a gradual network recovery through activity-dependent neuronal plasticity or adaptability induces the antidepressant effect [26, 27]. These intense investigations on neurotrophic factors have led to the formulation of the neurotrophic hypothesis of depression, which will continue to be a critical event in the pathophysiology and therapy of major depression. 

Recent findings suggest that early childhood events and adult stress produce neurodegenerative changes in the brain that can eventually lead to breakdown of the neuronal networks regulating mood [28, 29]. Therefore, genetic and/or functional defects in the critical neural networks may be the fundamental event of the major depression. In addition, environmental stimulations such as stress may eventually cause the major depression disorder.

## 3. Molecular Features and Functions of CART

The CART or CARTPT (CART prepropeptide) gene was discovered from rat brain in 1995 by PCR differential display in a search for gene transcripts in the striatum acutely up-regulated by psychostimulants [7, 10]. CART is a unique gene without significant homologues, and well conserved across species—from fishes to mammals and humans. Its human chromosome location is 5q13.2 (GenBank accession number and locus: NG_015988; homo sapiens CART/CARTPT mRNA accession number: NM_004291). In the rat brain, the primary CART transcript is differentially spliced and the two mRNAs encode CART prepropeptides of either 116 or 129 amino acids (a.a.) [7]. The leader sequence of CARTPT consists of 27 a.a. and mature CART peptides, therefore, contain either 89 or 102 residues [7]. Only the former form of CART (89 a.a. long) is present in human tissue [30]. CART peptide processing is tissue dependent and two forms predominate in the rat brain: CART (42–89) and CART (49–89) [31]. These C-terminal ends of CART, consisting of 48 or 41 amino acid residues (long form and short form, resp.) and 3 disulphide bonds, are thought to constitute a biologically active part of the molecule. Many studies subsequently showed that both of these peptides are active, and they may have different actions. 

As shown in the first CART paper [7], CART is expressed in the striatum, as well as its ventral extension, the nucleus accumbens (NAc), further CART peptide immunoreactivity are also located in a number of hypothalamic structures involved in the control of feeding behavior, stress response, and body homeostasis, such as the lateral hypothalamic area, the paraventricular and arcuate nuclei [10, 30, 32–34]. Consistent with these anatomical observations, a number of groups have shown that intracerebroventricular (ICV) administration of CART peptides inhibits food intake in rats and mice [10, 34]. CART knockout mice displayed increased body weight at the age of 40 weeks or when offered a high caloric diet, further indicating that CART is required for maintaining normal body weight and energy homeostasis [35, 36]. 

CART contains three disulfide bridges in its C-terminal part. This unique structure is not known to exist in other peptides/proteins [37], and this impact structure may increase CART's permeability across most cellular membranes. In fact, a previous report showed that CART readily passes through the blood-brain-barrier after intravenous injection, and the entry of CART into brain is rapid and not inhibited by excess CART or leptin [38]. This feature may facilitate CART use in animals (including primates) and humans in the future. 

Recently, using primary neuronal cell culture, we found that CART has an important transcription activity when fusing the CART C-terminal to the yeast Gal4 DNA-binding domain [39]. This function may explain its regulation role in the expression of several genes, including BDNF. However, the detail molecular mechanisms as well as the relationship between this transcription activity and behaviors need to be further elucidated.

## 4. Depression and CART in Gender Difference

Although MDD affects men and women of all ages, races, and economic levels, women are at a significantly greater risk than men to develop major depression. Studies show that episodes of depression occur twice as frequently in women as in men [5, 40]. The mechanisms underlining this phenotype are not fully understood. Sex differences in the structure or function in stress-responsive systems may play a role in female vulnerability to the disease. It has been shown that the major brain norepinephrine- (NE-) containing nucleus, locus coeruleus (LC), is more sensitive to stressors and to the stress-related neuropeptide, corticotropin-releasing factor (CRF) in female compared to male rats [41, 42]. Further, the greater dendritic extension and complexity seen in females predicts a higher probability of communication with diverse afferents that terminate in the peri-LC. This may be a structural basis for heightened arousal in females, an effect which may, in part, account for the sex bias in incidence of stress-related psychiatric disorders [43].

The nonpreganglionic Edinger-Westphal nucleus (npEW) in midbrain has also been implicated in regulation of the stress response. CART and BDNF are sex-specifically involved in the stress response too, and are present in the human and rat npEW [44]. Immunocytochemistry and in situ hybridization have shown that BDNF, CART and the estrogen receptor beta (ERbeta) are colocalized in the npEW. Quantitative immunocytochemistry revealed a 16% lower number of BDNF-immunoreactive neurons, and 19% lower CART-immunoreactivity in females compared to males [44]. Furthermore, chronic stress increased the amount of CART and nesfatin-like immunoreactivity in both males and female rats [45], indicating a firmed adaptive change in these long term-stressed animals. In a mouse study, acute (restraint) stress stimulates the general secretory activity of these npEW neurons as determined by increased presence of Fos, and the production of CART, urocortin-1 (Ucn1) mRNAs have been significantly increased compared to controls [46]. Therefore, CART, Ucn1 and nesfatin-1/NUCB2 are specifically involved in the response of npEW neurons to stress. Considering the fact that CART, Ucn1, and BDNF are coexpressed in the npEW with ERbeta, and their protein expression differs between males and females, the authors proposed that the functioning of the npEW may contribute to the sex differences that exist in stress sensitivity [44, 46].

Further, stress exposure and diet have differential effects on CART and BDNF that are sex dependent [47]. Chronic mild stress, a widely recognized animal model of depression, showed that gene for neuropeptide Y was upregulated in female rats purely in response to stressors, whereas genes for CART, BDNF, and arginine vasopressin (AVP) in males, and leptin in females, exhibited a significant response to the interaction between stressors and diet. Every affected gene showed a different pattern of expression in males and females. Therefore, this study further supports a neurobiological basis for differences in the affective state response to stress in males and females [47].

The CART levels in mesolimbic brain areas have also been analyzed [48]. The specific expression of the CART transcript in the ventral striatum, the amygdaloid complex, and the amygdala hippocampal area, strongly suggests an involvement of CART in emotion, motivation, and reward [48, 49]. Furthermore, it has been shown that CART expression is regulated by stress in a regionally and time-specific manner and that CART is regulated by corticosteroid actions in the hippocampus [49]. Also interestingly, it has been found that a gender difference in the basal CART mRNA expression that was restricted to the nucleus accumbens shell. Male rats expressed higher levels of CART mRNA in this region than did female rats [48]. 

Such different features between female and male in brain may be due to gonadal hormones and the interaction between hormone, stress, and brain [40, 50–52]. Gonadotropin-releasing hormone (GnRH) is the pivotal hypothalamic hormone regulating reproduction and related behaviors [53]. As mentioned above, CART has a transcription activity in neuron cells, it has been shown that CART (42–89) is also involved in regulation of GnRH in hypothalamus [54]. The release of GnRH is intricately related to sex steroid hormones, especially neuroprotective estrogen [55–57]. Recent study showed that estrogen can promote stress sensitivity in a prefrontal cortex-amygdala pathway [58]. On the other hand, estrogen can increase CART production in cortical neurons [59]. Further CART increases expression of a key neurotrophic factor BDNF, which mediates neuronal growth, neuroprotection, and synaptic modulation in hippocampal neurons [60]. BDNF is also associated to the pathology of depression and is thought to be a key target in the treatment of major depression [61, 62]. Taken together, CART and its association factors such as BDNF, estrogen/estrogen receptor, may be involved in the gender different mechanism in stress and depression. To date, however, CART serum levels in normal human and patients with mental diseases (including MDD), in particular the relationship between CART and gender have not been reported.

## 5. Antidepressant Action of CART

### 5.1. Antidepressant Action of CART in Animal Studies

CART peptide and mRNA are present in brain regions that are associated with depression, including the hippocampus, the locus coeruleus, parts of the midbrain raphe nuclei, the amygdale, and the hypothalamus [7, 48, 49, 63]. It was recently demonstrated that CART mRNA is downregulated in the frontal cortex of rats that have been subjected to a chronic mild-stress paradigm, which is an animal model of depression [20], indicating that chronic mild-stress inhibits normal CART expression in the frontal cortex. However, another recent report provided evidence for the CART peptide being significantly increased in the periaqueductal grey in a genetic rat model of depression [64]. This increased expression of CART may represent an adaptation to the transgenic depression model, it has been considered that the adaptation changes may be the key to treatment of some diseases, especially stress-related disorders [65].

 To investigate the effect of CART peptide on depression, two depression-like behavioral rat models (i.e., socially isolated and olfactory bulbectomized, OBX) were used. Administration of CART (54–102) into the lateral ventricle (50–100 ng) or central nucleus of amygdala (CeA) (10–20 ng) caused significant decrease in immobility time in the forced swim test (FST) without influencing locomotion. This suggests that the CART peptide may have an antidepressant-like effect [66]. Social isolation as well as OBX models were undertaken to produce depression-like conditions. Although isolation reared (6 weeks) rats showed significant increase in immobility time in FST, OBX animals exhibited hyperactivity (increase in the ambulation, rearing, grooming, and defecation scores) on day 14 in the open-field test. The isolation- or OBX-induced depression-like phenotypes were reversed following acute or subchronic treatment of CART, respectively, given via ICV and intra-CeA routes. Drastic reduction in CART-immunoreactivity was observed in most cells in PVN, arcuate and Edinger-Westphal nuclei of the socially isolated and OBX animals. Although the fibers in the paraventricular nucleus (PVN) showed variable response, those in the arcuate nucleus (ARC) and prefrontal cortex did not change. The CART-immunoreactive fibers in the locus coeruleus also showed highly significant reduction. However, dramatic increase in CART-immunoreactive fibers was noticed in the CeA in both experimental models. These data demonstrated the antidepressant role of CART when using lower doses (even 10 ng), and underscores the important fact that the endogenous CART system might play a major role in mediating symptoms of depression. Since there are several biologically active forms of CART, and they may have similar or different effects on the depression model, other main forms of CART should also be tested in the future. This is still interested in the field. 

Similarly, as discussed above, CART expression levels were significantly increased in the rat NAc after electroconvulsive therapy, which also suggests a beneficial role for CART as an antidepressant treatment [67]. It is important to mention that in these antidepressant studies and our neuroprotection study [12], the dosage of CART peptide was much lower than that of food intake studies [34]. This indicates that CART functions primarily as an antidepressant at physiological concentrations, which is further supported by other studies both in vitro and in vivo [59, 60]. Paradoxically, an anxiety-like effect has also been observed in animal studies as discussed below (Section 7). Multiple factors such as dosage, structure and size of the CART peptide used may play a role in causing anxiety. From this point, anxiety-like behavior may be a side effect of CART peptide usage, but this needs more studies for clarification. Then, the CART molecule (structure, size, and dose) can be selected or further modified to improve its efficacy as an antidepressant, with minimal side effects.

### 5.2. Antidepressant Action of CART in Human

There is no report regarding CART clinic trial on human diseases so far. Recently, it was hypothesized that the frequent co-occurrence of mood disorders and obesity may be characterized by interconnected pathophysiology [68]. Therefore, if neuropeptide CART is involved in both disorders it may be useful for the individuals suffering the combined disorders. Animal experiments have discovered a connection between this peptide and mood disorder and obesity: social isolation might downregulate the hypothalamic CART-containing system, which in turn may lead to increased food intake and body weight [47, 69, 70]. Interestingly, the hypothesized connection of the CART system to depression in humans derives from a study of an Italian family with early-onset obesity and a missense mutation in the CART gene [71]. Just as obesity co-segregated with the mutation among the family members, high levels of both depression and anxiety were found in family members with the mutation [72]. Although the number of subjects in this study was small, the results are intriguing. This is the first suggestion that the CART gene may be involved in depression and anxiety in humans. It is as yet unclear whether the anxiety and depression are consequences of other problems arising from the mutation, or whether they are a more direct consequence of the lack of normal CART peptides [10, 71]. Even though the detail mechanism is unclear, these observations strongly indicate the importance of CART gene and the normally encoding products on both body weight control and mental stabilizations. In other words, normal CART peptides, but not the mutant, have a potential antidepressant role in humans.

## 6. Mechanisms of CART Antidepressant

### 6.1. Regulation of HPA System

Although the cause of depression is complex, it is often described as a stress-related disorder. The stress response is mediated by the hypothalamic-pituitary-adrenal (HPA) system. Activity of the corticotropin-releasing hormone (CRH) neurons in the hypothalamic PVN forms the basis of the activity of the HPA-axis. The CRH neurons induce adrenocorticotropin (ACTH) release from the pituitary, which subsequently causes cortisol release from the adrenal cortex. The HPA-axis is considered to be the “final common pathway” for a major part of the depressive symptomatology [73]. 

There is evidence suggesting a role for CART in the regulation of the HPA-axis. First, CART peptides are present in each of the three components of the HPA-axis [74, 75] and in blood [10, 76, 77]. This distribution suggests many roles for the CART system in stress response, in addition to its role in feeding, endocrine regulation and circadian rhythm. Second, physiological and pharmacological studies showed that central administration of CART dramatically increases phosphorylation of cAMP response element binding protein (CREB) in cell nuclei in corticotropin-releasing hormone-producing neurons in the hypothalamic PVN in fasted as well as fed rats at 10-min postinjection, particularly in the medial parvocellular subdivision of the PVN [78]. CREB is an important transcription factor, which responds to environmental signals, CREB phosphorylation is necessary for neuron survival. Furthermore, ultrastructural analysis revealed that CART establishes axosomatic and axodendritic contacts with CRH neurons in the PVN [78].

CART is capable of increasing the gene expression of both CRH and thyrotropin-releasing hormone (TRH) in hypophysiotropic neurons. Additionally, CART-containing axon terminals establish synaptic relationships with hypophysiotropic CRH and TRH neurons. Therefore, it is possible that through its many functions, CART may signal the HPA-axis by more than one pathway. For example, CART regulates the activity of the HPA-axis through a corticotropin-releasing factor- (CRF- or CRH-) dependent central mechanism [79], and partly through a direct interaction with the pituitary corticotropes [80, 81]. Interestingly, the stimulating effect of CART on serum ACTH concentration was observed only 30 min after ICV injection. However, CART stimulated corticosterone release at 10, 30, 60 min after ICV injection [81]. This observation suggests that CART has an acute effect as well as a long-lasting cascade effect on adrenal gland through direct (local adrenal only) and/or indirect (hypothalamus-pituitary-adrenal cascade) pathway. Hence, the translation of these interactions in HPA-axis system into identifying a novel therapeutic target is promising [11, 82, 83].

### 6.2. Limbic System, Dopamine, and Serotonin Transmitters

The limbic system, which is thought to have a role in regulating emotion, is very important for modulating affect and anxiety. It is also an important target for contemporary antidepressants [10, 84]. CART peptides are expressed in several parts of the limbic system, including the central and basomedial nuclei of the amygdala, the bed nucleus of the stria terminalis, and the hippocampus [7, 85]. 

**Figure 1:**
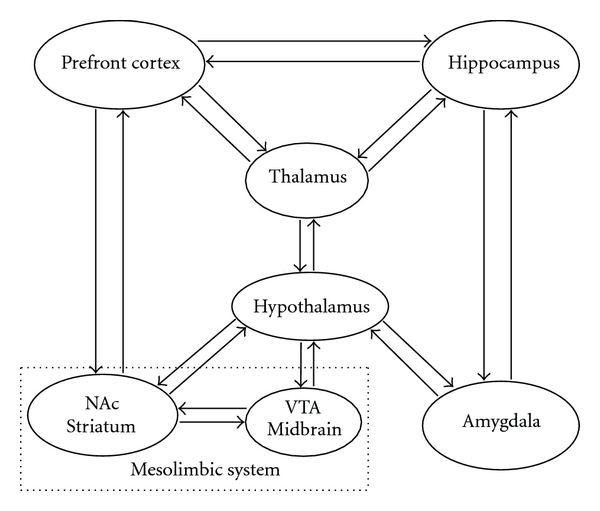
CART is highly expressed in the most structures of the limbic system which is known to be the seat of the emotions. This simplified diagram depicts the major limbic circuitry and main pathways that may mediate the antidepressant role of CART. The midbrain ventral tegmental area (VTA) and the connection with the nucleus accumbens (NAc) in the ventral striatum also form the mesolimbic dopamine pathway (square with dotted line). Both CART mRNA and peptide are abundant in the depicted brain regions. In human brain, the highest CART mRNA expression levels were found in hypothalamus and thalamus [86].

The mesolimbic dopamine system is most often associated with the rewarding effects of food, sex, locomotor activity, and drug abuse [87, 88]. The NAc (ventral striatum) and its dopaminergic input from the ventral tegmental area (VTA) form together the mesolimbic dopamine system (Figure 1) [87]. Given the prominence of anhedonia, reduced motivation, and decreased energy level in most individuals with depression, it has been proposed that the NAc and VTA contribute importantly to the pathophysiology and symptomatology of depression and may even be involved in its etiology [87, 89]. 

The major limbic structures, including the amygdaloid complex, dentate gyrus of the hippocampus, medial prefrontal cortex, hypothalamic mammillary and supramammillary nucleus, and NAc, all express CART mRNA and protein (Figure 1), suggesting a potential role for CART in the treatment of psychiatric disorders in which stress responses, mood and reward processes are involved [10, 30, 48, 49, 86–90]. The dopaminergic system of the NAc operates within the limbic system, which is closely related to mood. CART has been shown to increase dopamine and serotonin turnover in the NAc [91, 92], and both of these neurotransmitters are important mood regulators. Therefore, the homeostatic role of CART via dopaminergic system in these regions may also prove to be beneficial in the treatment of major depression disease. It has been shown that CART increases phosphorylation of CREB protein in the hypothalamus [78]. Interestingly, use of electroconvulsive therapy, a well-known antidepressant and antipsychotic treatment, increased CART mRNA and protein levels in the rat NAc. This increase of CART mRNA was also accompanied by an increase in phosphorylation of CREB [67], which indicates the potential important mediating role of CART in the therapeutic effects of electroconvulsive shock. 

CART was also found to alter the activity of the striatal noradrenergic and corticostriatal and hypothalamic serotoninergic system and enhance their effect on the central dopamine system, despite the role of these effects not being completely understood [93–95]. To investigate the effect of CART peptides on extracellular 5-hydroxytryptamine (5-HT, serotonin) in NAc and the dorsal raphe nucleus (DRN)—which contains the most serotonergic neurons projected to the forebrain, including the NAc—a microdialysis approach was used in freely behaving rats [92]. Reverse infusion of CART 61–102 (short form) in the DRN produced a concentration- (10–100 microM) dependent increase in 5-HT in the DRN. Similarly, CART 62–76 (10–100 microM) infused into the DRN and NAc elevated 5-HT in the DRN and NAc, respectively. Thus, CART increases extracellular 5-HT in both the DRN and NAc. In addition, infusion of higher dose CART 62–76 (100 microM, super short form) in the DRN produced a significant increase of 5-HT in the NAc. This suggests the possibility of CART receptors being responsible for the depolarization-dependent release of 5-HT. In summary, these results suggest that CART peptides may have an antidepressant effect through increases in extracellular 5-HT [92]. On the other hand, the ICV administration of IgG antibodies against CART in rats upregulates mu and serotonin 5-HT (2A) receptor in the hippocampus and caudate. This finding demonstrates that endogenous CART peptides in the cerebrospinal fluid may exert regulatory effects on serotonin system and other signaling in the brain [96]. Further study of depression animal model(s) will be very interesting given the preliminary evidence of CART levels being increased in two animal models of depression and anxiety [64].

### 6.3. Energy Homeostasis and Mitochondrial Booster

To determine the mechanisms of CART's multiple functions, the field has been faced with the challenge of identifying the CART receptor and interaction partners [10]. Although the CART-specific binding sites in culture cells and the hypothalamus have been characterized, the receptor(s) for CART have not yet been identified [10]. Recently, the first interaction protein for CART, the mitochondrial succinate dehydrogenase B (SDHB) [12], was identified by a yeast two-hybrid system. Related to this finding, CART also stimulates SDH activity and increases ATP level and neuron survival in normal and pathological (i.e., ischemia) conditions. Furthermore, this action is efficient at lower CART concentrations [12]. Succinate dehydrogenase (mitochondrial respiratory complex II) is a key member of both the Krebs tricarboxylic acid cycle (TCA cycle) and the aerobic respiratory chain; both of which are critical for intermediary metabolism and energy production. The observations from CART knockout mice, in which the normal relationship between CART and mitochondrial SDH disappeared, clearly indicate that CART is involved in the physiological regulation of feeding and energy homeostasis (35, 36). Given its role as a SDH interaction partner and mitochondrial booster, CART may be suitable for treatment of some debilitating diseases, including mood disorders (MDD), obesity/diabetes, and neurodegenerative diseases. 

Furthermore, CART is also colocalized with the melanin-concentrating hormone (MCH) and orexin-containing neurons in the hypothalamic circuits that control the energy homeostasis, which can affect the vegetative function in patients suffering major depression [97]. CART may co-ordinate with these factors during regulation of the energy homeostasis, including in the animal depression models as well as MDD patients.

### 6.4. CART Increases Other Neurotrophic Factors Particularly BDNF

Neurotrophic factors, as survival factors, are critical regulators of the formation, development and plasticity of neuronal networks in the nervous system [28, 98]. Increasing evidence suggests that neuronal plasticity and neurogenesis play an important role in the recovery from depression. Antidepressant treatments increase the expression of several molecules, which are associated with neuronal plasticity and neurogenesis, in particular the neurotrophin BDNF and its receptor tyrosine kinase B (TrkB). BDNF is broadly expressed in mammalian and human brain, including the hippocampus and prefrontal cortex. BDNF together with TrkB plays an important role in development, neural regeneration, synaptic transmission, synaptic plasticity, and neurogenesis [27, 28, 99–101]. Therefore new agents capable of enhancing BDNF levels may lead aid the development of novel therapeutic drugs for treatment of MDD and other mental diseases. Interestingly, CART is highly expressed in rat hippocampus and can promote the survival and differentiation of neurons in primary hippocampal cell cultures. This neurotrophic role is mediated by increasing BDNF expression, and blocked by TrkB antibody [60]. The precise mechanism underling this action is unclear. It has been shown that CART activates the extracellular signal-regulated kinase (ERK) pathway in pituitary-derived AtT20 cells via putative G-protein coupled receptors [102], and this increase in the phosphorylation of ERK was confirmed in primary cortical neurons [58]. The activation of the ERK family of MAP kinases promotes survival of neurons eventually [103]. CART also significantly increases the levels of phosphorylated ERK and phosphorylated N-methyl-d-aspartate (NMDA) receptor NR1 subunit in the spinal cord [104]. More directly, CART has a putative transcription activity in neuronal cells, once CART peptide enters to the cellular nuclei, CART can induce transcription of target genes [39]. Among these CART-associated genes/proteins, in turn, transcription factor CREB regulates genes whose products are essential for prolonged neurotrophin-dependent survival of neurons [105, 106]. 

In addition, inhibitory transmitter *γ*-aminobutyric acid (GABA) is another crucial element in the neuroendocrine and modulatory systems in the hypothalamus, and GABA receptors are the targets for many psychotropic drugs including antidepressants [107]. CART and the subunit of the GABA_A_ receptors are coexpressed in the hypothalamus [107, 108] and in the midbrain [109]. It has been suggested that the CART peptides may be used as a cotransmitter in a subpopulation of intrinsic GABAergic terminals in the shell of NAc, as evidenced by the intense immunoreactivity in dense-core vesicles forming a symmetrical synapse in a primate study [110]. Although it is not known how the CART peptides modulate the release of GABA from intrinsic striatal terminals, the interaction between CART and the GABA receptors might provide some critical clues regarding the mechanism of antidepressants.

## 7. Concerns: Anxiety-Like Action for Some CART Peptides

There is evidence for an involvement of CART peptides in anxiety-like behavior [7, 10, 111]. It was reported that in rats and mice, ICV injection of small CART peptide fragments (89–103, super short form, 15 a.a.) increased anxiety-like behavior in the elevated plus-maze, and ICV administration of CART (82–103, super short form, 22 a.a.) (0.04–5.0 nmol) did not inhibit water intake and did not affect spontaneous locomotor activity. The authors suggested that CART might be an anxiety/arousal peptide as primary action, and the effects on food intake may be secondary due to anxiety/arousal [112]. 

In another study, ICV administration of long form CART 55–102, but not short form CART 62–102 (or CART 49–89 in humans), increased anxiety-like behavior as measured in the elevated plus-maze and in a social-interaction test [113]. Interestingly, CART 49–89 in the biologically active dose range had no effect on time spent in open arms of the elevated plus-maze. Therefore, two biologically relevant forms of CART peptide can be distinguished in vivo based on their potential to cause anxiety in mice. These results suggest that different CART peptides may have different roles, and that there might be more than one CART receptor. Thus, we propose that the short form of CART peptide (CART 49–89) may be more preferred as a potential antidepressant agent. 

## 8. Conclusions and Future Directions

The etiology of major depression disorder (MDD) is still unclear. It is likely a complex of multiple factors, including genetic, environments and psychosocial factors. Recent studies on the involvement of neurotrophic factors in the regulation of mood disorders and antidepressant effects have led to the formulation of the neurotrophic hypothesis of depression [26–29]. The currently available evidence from molecular feature, gene expression, mutation analysis and translational studies suggests that new neurotrophic factor CART (or CARTPT, cocaine- and amphetamine-regulated transcript prepropeptide) has possible therapeutic implications in the treatment of major depression and other mood disorders and comorbid obesity. CART has the potential to play an important role in the treatment of major depression through several mechanisms.

1. CART performs an antidepressant effect through the regulation of certain cascades in the signal transduction pathway by modulating kinases, other neurotrophic factors and neurotransmitters such as BDNF, serotonin, dopamine, and GABA, respectively, resulting in increased neuronal survival and an alteration in the synaptic plasticity positive to a treatment of depression.
2. CART also enhances mitochondrial activity by acting as a partner of the key mitochondrial enzyme SDH, therefore CART increases cellular energy in the treatment of debilitating depression.
3. CART may regulate the HPA-axis feedback loop through the regulation of corticotropin-releasing factor (CRF or CRH), some pituitary corticotropes and adrenal hormones, at least partly via its transcription activity.

It is important to continue investigating the roles of CART in the treatment of depression (including animal models of depression), which will undoubtedly lead to an enhanced understanding of the molecular actions of antidepressants. It should be noted that CART short form may provide the most promise for further clinical trials, and lower doses of CART will have reduced side effects. Moreover, preliminary evidence of some promising roles of CART in the regulation of serotonin, noradrenaline, and dopamine—all of which are essential players in the mechanism of antidepressants—may provide important clues in the development of novel antidepressants.

In conclusion, CART peptide has a novel antidepressant function in mammals and humans, and CART or its derivatives may be promising antidepressant treatments.
